# From taxonomic deflation to newly detected cryptic species: Hidden diversity in a widespread African squeaker catfish

**DOI:** 10.1038/s41598-019-52306-2

**Published:** 2019-10-31

**Authors:** Dagmar Jirsová, Jan Štefka, Radim Blažek, John O. Malala, David E. Lotuliakou, Zuheir N. Mahmoud, Miloslav Jirků

**Affiliations:** 1 Faculty of Science, University of South Bohemia, Branišovská 1760, 370 05 České, Budějovice Czech Republic; 2 Institute of Parasitology, Biology Centre, Czech Academy of Sciences, Branišovská 31, 370 05 České, Budějovice Czech Republic; 3 Institute of Vertebrate Biology, Czech Academy of Sciences, Květná 8, 603 65 Brno, Czech Republic; 4 Department of Botany and Zoology, Faculty of Science, Masaryk University, Kotlářská 2, 611 37 Brno, Czech Republic; 5 Kenya Marine and Fisheries Research Institute, Lake Turkana Station, P.O. Box 205, 30500 Lodwar, Kenya; 6 Department of Zoology, Faculty of Science, University of Khartoum, 111 15 Khartoum, Sudan

**Keywords:** Phylogenetics, Ichthyology

## Abstract

Cryptic genetic diversity and erroneous morphological species determination represent frequent problems in biodiversity research. Here, examination of 138 specimens of *Synodontis* (Mochokidae, Siluriformes) from the Nile River and Lake Turkana revealed the presence of both *S*. *schall*-like and *S*. *frontosus*-like morphotypes, with a phenotypic gradient between them. We concluded phylogenetic and population genetic analyses based on two mitochondrial and one nuclear marker including 131 *coxI* (565 bp), 96 *cytb* (973 bp) and 19 *RAG2* (896 bp) sequences from the Nile-Turkana population, plus additional GenBank data of *Synodontis* spp. Whilst nuclear data were inconclusive, mitochondrial sequences suggested that both morphotypes and intermediate forms are conspecific. The results imply probable synonymy of *S*. *frontosus* with *S*. *schall*. Conversely, a strong biogeographical signal was revealed among widely distributed and supposedly conspecific *S*. *schall*-like catfish of the Nilo-Sudanian ichthyological province. *Synodontis schall* sensu stricto (=Eastern clade), as defined by type locality in the Nile, is apparently restricted to the eastern part of the Nilo-Sudanian ichthyological province (e.g. Nile, Turkana, Chad). *Synodontis schall* Western clade (Senegambia, Niger, Chad) most probably represents a cryptic taxon, unrecognized thus far due to the absence of distinctive morphological differences.

## Introduction

Determining and identifying independent lineages and species is critical not only for taxonomy, but also for understanding the processes leading to diversification and the origin of population structure. Due to the extensive sampling, discovering cryptic species within the scope of population genetic studies is not a rare phenomenon^1–3^. Traditionally, cryptic species/diversity in fishes is mostly discovered in highly variable taxa, especially in the species rich lineages of ray-finned fishes, such as the neotropical Characidae^4,5^ or Cichlidae^6,7^. Cryptic diversity was also found - albeit less frequently - in other lineages, e.g. spiny eels^8^ and Neotropical^9–11^ and African catfish^12,13^, including the family Mochokidae^14–16^. Generally, the main contributing factor to the undiscovered cryptic diversity of some species is the lack of clear distinguishing morphological features in combination with the absence of relevant molecular data^3^. This not only underestimates the species richness but also limits our understanding of species geographical distribution.

Within the scope of research on fish parasites in the Nile River and Lake Turkana, we examined a total of 138 *Synodontis* specimens. Two of three *Synodontis* spp. in our sample, *Synodontis nigrita* Valenciennes, 1840 and *Synodontis serratus* Rüppell, 1829 (both absent in Lake Turkana) could be readily morphologically determined. The third species *Synodontis schall* (Bloch et Schneider, 1801) showed deviation from both, the data in the literature, as well as the differential diagnosis and neotype proposed recently by Musschoot & Lalèyè^17^. Among *Synodontis* spp. occurring in the Nile and Turkana basins, *S*. *schall* might be confused with the morphologically very similar *Synodontis frontosus* Vaillant, 1895. According to the literature, *S*. *schall* is common and abundant in both the Nile River and Lake Turkana^18–21^. Although the two species might co-occur in both basins, *S*. *frontosus* is considered absent in Lake Turkana itself, reportedly being restricted to its main source, the Omo River^22^. Diagnostic features of both species are similar and partly overlapping, therefore, distinguishing between the two in the field is difficult.

Resolving the species status of *S*. *schall* is important as the species has been used as a model in parasitological research^23^. Therefore, we decided to base the taxonomical assignment of our Nile-Turkana *S*. *schall* specimens on a multidisciplinary approach, particularly on molecular identification with an assessment of selected morphological traits. Herein, we assess the identity of *S*. *schall*-like catfish from the Nile and Turkana basins by means of phylogenetic and haplotype analyses of a representative set of *Synodontis* spp. *coxI*, *cytb* and *RAG2* sequences, revealing a previously unrecognized biogeographical pattern. To the best of our knowledge, this is the first confrontation of morphological features traditionally used for determination of *S*. *schall* and similar Nilo-Sudanian catfishes with molecular data to aid proper distinction of these catfishes of high significance from both an ichthyological and regional fisheries perspective.

## Material and Methods

### Study group

The Mochokidae (Actinopterygii, Siluriformes) is an endemic African family of freshwater catfish containing 211 species in nine genera^24^. With 131 nominal species currently recognized, *Synodontis* Cuvier, 1817 is the most speciose mochokid genus widely distributed in all African ichthyological provinces except for Maghreb and South Africa^18,21,25^. Despite numerous studies based on genetic markers^26–28^ morphology remains the main classification tool for identification of *Synodontis* spp.^17,18,29^. Several molecular phylogenetic studies were published during the last 13 years which elucidated general biogeographic patterns and relationships within the genus^26–28,30,31^. *Synodontis schall* is traditionally considered widespread throughout the Nilo-Sudanian ichthyological province from the Atlantic to Indian Ocean^17^. Its widespread distribution, abundance and easy harvesting make it one of the most important subsistence fish species in Sub-Saharan Africa north of equator and along the whole Nile valley^28,32–34^. Importantly, this ecologically plastic species^28^ is less likely to be affected by climatic changes and anthropogenic drainage alterations, making it a prospective source for fisheries in the future^35^. Despite its significance for fisheries and a long taxonomical history, morphology-based distinction between *S*. *schall* and similar species such as *S*. *frontosus*, remains unclear.

### Sampling and fish determination

Sampled fish were obtained in seven different localities in the Nile and Turkana basins (*S*. *schall* n = 120, *S*. *nigrita* n = 8, *S*. *serratus* n = 10, Total: 138; see Table 1 for details). The two Nile localities cover both the White (Kostí) and Blue Nile (Sennar). The five Turkana localities cover the salinity gradient present in the lake, i.e. the freshwater Omo River delta (Todonyang) and the saline (brackish) main part of the lake (central Turkana saline: Kalokol, Central Island; central Turkana freshwater refugium: Kerio River delta; south Turkana: El-Molo Bay). Fish examined were obtained in fish markets and/or from fishermen and processed under supervision of Kenya Marine and Fisheries Research Institute (KMFRI) authorities. Fish that were still alive when obtained were euthanized by dorsal pithing (spinal cord and blood vessels cut immediately behind the head), a method congruent with Kenyan, Sudanian, Czech and European legislation. Fish were destined for human consumption; therefore, no permission was needed to collect these fish. A small piece of liver tissue from each individual was cut off and preserved in 96% ethanol for molecular analyses. Fish were photo-documented and their standard length was measured. Their mouthparts including tooth plates and barbels were removed and preserved in 4% formaldehyde for further morphological examination.

**Table 1:** Sampling details.

Locality	Coordinates	n		
**Nile River**		*S*. *schall*	*S*. *nigrita*	*S*. *serratus*
White Nile in Kostí	13.1722 N, 32.6722 E	17	6	7
Blue Nile in Sennar	13.5436 N, 33.6366 E	21	2	3
**Lake Turkana**				
El-Molo Bay - saline	2.8322 N, 36.6958 E	45	absent	absent
Kalokol - medium salinity	3.5586 N, 35.9158 E	2	absent	absent
Kerio River delta – freshwater	2.9873 N, 36.1628E	8	absent	absent
Central Island - medium salinity	3.4958 N, 36.0403 E	2	absent	absent
Todonyang, Omo delta - freshwater	4.4517 N, 35.9439 E	25	absent	absent
Totals (n)		120	8	10

Upon field examination, all fish were determined based on the following combinations of qualitative diagnostic features: *S*. *nigrita*: body, head and fins with black spots, inner mandibular barbels bearing thick and tuberculate ramifications, maxillary barbels with a well visible broad black membrane, short and small adipose fin; *S*. *serratus*: maxillary barbels with a very large light-colored membrane, humeral process pointed, non-keeled, distinctive serrations on both edges of pectoral spine, prolonged rostrum^20,21^; for *S*. *schall*, see Table 2 and Results and Discussion sections. In congruence with taxonomic literature, the species identification was based on visual inspection of qualitative diagnostic features only as no relevant morphometric diagnostic traits are available for other *Synodontis* spp.

**Table 2:** Distinguishing features of *Synodontis schall* and *Synodontis frontosus*; data obtained within the scope of this study compared with the following literature: ^1^Hopson (1982), ^2^Bailey (1994), ^3^Paugy & Roberts. (2003), ^4^Musschoot & Lalèyè (2008). Note the intermediate states in the present Nile-Turkana sample and its overlap with literature data for both *S*. *schall* and *S*. *frontosus*.

Assessed diagnostic features	This study - Nile-Turkana (figure references given)	Literature	
	*S*. *schall* + *S*. *frontosus* + intermediate morphotypes	*S*. *schall*	*S*. *frontosus*
Humeral process ventral keel	usually present, more or less pronounced (1d–f,h), sometimes absent (1f,g)	non-keeled^1^ vs. slightly-keeled^3^ vs. usually non-keeled^4^	non-keeled^1,3^
Humeral process surface sculpturing	granulose with striated ventral keel	striated^1^ vs. granulose^3^	granulose^1,3^
Humeral process shape	upper margin straight (1d), concave (1e) or wavy (1g), lower margin straight (1d,g,h) or convex (1e,f), posterior tip sometimes curved (1f)	pointed^3^, upper margin straight or a little concave, lower margin straight or a little convexe^4^	deep, pointed^3^
Mandibular teeth	26–46 (21–40 in single row)	23–33^1^, 24–32^2^, 24–39^3^, 18–32^4^	36–48^1,3^, 33–48^2^
Pectoral spine anterior serrations	distinct, fine, sharp (1i), or barely discernible (1j)	Fine, sharp, obvious^1,3^	barely discernible^1,3^
Maxillary barbel membrane	none, rudimentary, or distinct dark-colored (1k-m)	none^1,3^ vs. none or hardly visible^2,4^	distinct dark-colored^1–3^

### DNA extraction, PCR and sequencing

DNA was isolated using the phenol:chloroform:isoamyl alcohol (25:24:1) method described in Sambrook & Russell^36^. Two mitochondrial genes and one nuclear gene were used for phylogenetic analyses, cytochrome oxidase I (*coxI*), cytochrome b (*cytb*) and recombination activating gene 2 (*RAG2*), respectively (see Table S1 in Supplementary Data for a complete list of sequences and other details). Mitochondrial genes were chosen as they are known indicators of recently divided species and also because *coxI* and *cytb* are available in GenBank for a broad range of *Synodontis* spp. Due to the high abundance of *RAG2* for genus *Synodontis* in GenBank, this nuclear gene was selected to supplement mtDNA data in selected *Synodontis* spp. specimens – 23 representatives from sampling localities in the Nile and Turkana. Amplification conditions and PCR primers *FishF2* and *VF2_t1* for partial sequence of *coxI* gene (~800 bp) were taken from Ivanova *et al*.^37^. PCR reactions contained 12.5 μl of PPP Master mix, 10.5 μl of ultra pure water, 5 pM of each PCR primer and 0.5 μl of isolated DNA (100 to 170 ng). Published primer sequences (5′-GAC-TTGAAGAACCACCGTTG-3′ forward and 5′-TTTAGAATTCTGG CTTTGGGAG-3′ reverse) to amplify part of *cytb* (~1000 bp) according to Pinton *et al*.^27^. For *RAG2* (~1100 bp) amplification primers (forward 5′-TGY TAT CTC CCA CCT CTG CGY TAC C-3′ and reverse 5′-TCA TCC TCC TCA TCK TCC TCW TTG TA-3′) and reaction conditions followed those provided by Sullivan *et al*.^38^. Amplification reactions for *cytb* and *RAG2* contained the same PCR reagents and amount of DNA as mentioned above. All products were verified on a 1% agarose gel and purified using enzymes exonuclease I (Exo I) and shrimp alkaline phosphatase (SAP)^39^. Purified fragments were directly sequenced by Macrogen Inc. (Amsterdam, Netherlands) using the same primers used for PCR amplification.

### Phylogenetic, haplotype analyses and population genetic statistics

Obtained sequences were assembled and inspected for errors using the GENEIOUS Pro software package version 6.1^40^. In addition to our original sequences (131 *coxI*, 96 *cytb* and 19 *RAG2*, Table S1), we used GenBank sequences of *Synodontis* spp. (n = 115 *coxI* sequences from 77 *Synodontis* spp., n = 80 *cytb* sequences from 52 taxa, and n = 102 *RAG2* from 69 species) to interpret our results in a broader phylogenetic context (see Table S2 in Supplementary Data for complete list of sequences and additional information).

Alignments were constructed in MAFFT v. 6^41^ and corrected manually using the GENEIOUS Pro software package version 6.1. Best-fit models of molecular evolution were selected using AIC criteria in jMODELTEST v2.1.4^42^. Phylogenetic analyses were conducted for individual gene datasets plus a concatenated dataset of the two mtDNA genes (where data for both genes were available, see Tables S1 and S2). Phylogenetic analyses were performed using Bayesian inference (BI) in MrBayes v3.2.2.^43,44^ and maximum likelihood (ML) in PHYML v3.0^45^. BI analyses were performed under the same conditions for all datasets - using 5 million MCMC replications, with four chains and three independent runs. The following nucleotide substitution models were used: GTR + I + G for *coxI* data, GTR + G for *cytb*, HKY + I + G for *RAG2* and GTR + I + G for concatenated mt data. ML analyses were run using bootstrap analyses with 1000 replications and models mentioned above. Convergence for BI runs was estimated by TRACER v1.7^46^, ESS values were assessed and found to exceed 200 for all parameters. Trees computed for *cytb* were rooted using *Auchenoglanis occidentalis* (HM880259, KY483731, EU781895) and *Mochokus niloticus* (KY483655) as an outgroup. The remaining trees (*coxI*, *RAG2* and concatenated data) were rooted using GenBank sequences of *Microsynodontis* sp. (DQ886604 for *cytb* and HF565741 for *RAG2*). All trees were deposited in TreeBASE database (treebase.org) and are accessed under the following link http://purl.org/phylo/treebase/phylows/study/TB2:S23311.

Pairwise genetic distances were computed to assess the divergence of *S*. *schall* sequences and included sequences of a closely related taxon (*S*. *serratus*) and a distantly related congener (*S*. *nigrita*) were included for comparison. Due to the limited availability of the nuclear data, genetic distances were computed for mitochondrial data only.

To examine the level of population variability in the dataset, statistical parsimony networks based on pairwise differences were constructed for individual *S*. *schall* clades revealed by phylogenetic analyses using PopART v1.7^47^ with settings for TCS networks^48^.

Statistics of Haplotype diversity (Hd), *Fst* values, genetic distances and differentiation were calculated using DNASP v5^49^. Due to the limited number of GenBank sequences from the western part of *S*. *schall* geographic range, population statistics were performed only for *S*. *schall* samples obtained for this study (Nile and Turkana); data were split according to their resemblance to the *S*. *schall-like* and *S*. *frontosus-like* morphotypes (see Table S2).

## Results

### Morphology

**Figure 1:**
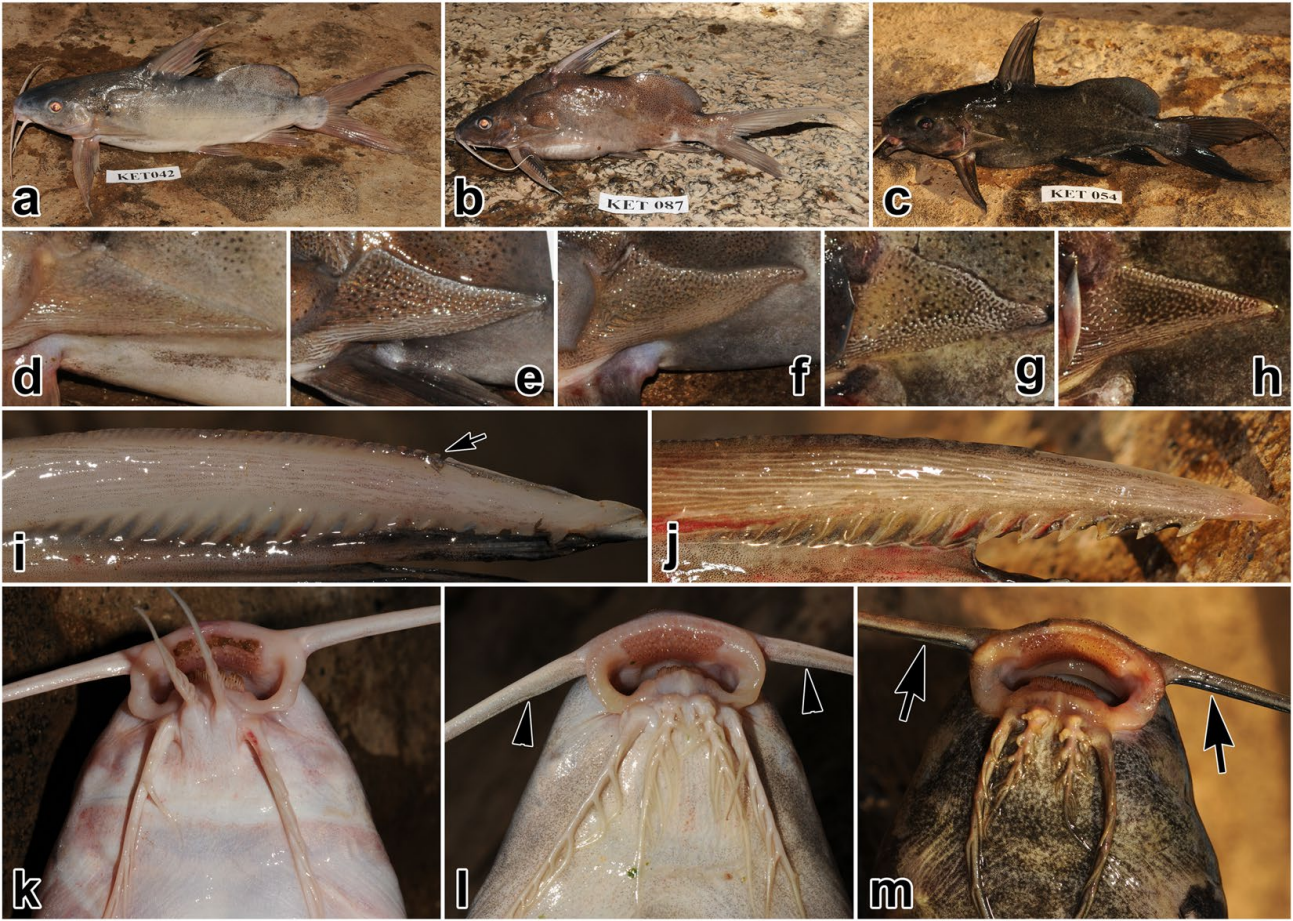
Qualitative morphological diagnostic features in live specimens of *Synodontis schall* from Lake Turkana, Kenya. Corresponding humeral processes, pectoral spines and mouthparts are shown for two specimens representing typical *S*. *schall* morphotype (**a**,**d**,**i**,**k**) and typical *S*. *frontosus* morphotype (1c,h,j,m); remaining pictures originating from different individuals show additional variation. (**a)** Todonyang (Omo River delta); (**b)** El-Molo Bay (southern Lake Turkana); (**c)** Todonyang; (**d–h)** Humeral process variants (see Table 2 for details); (**i)** Pectoral spine with well visible fine sharp anterior serrations (starting from small arrow towards left side of picture); (**j)** Pectoral spine with anterior serrations barely discernible (partly due to pigmentation); (**k)** Maxillary barbel without membrane; (**l)** Rudimentary slat-like form (arrowheads); (**m)** Distinct dark membrane (arrows). Note: Depicted specimens show numerous dark specks in various body parts, but specimens almost lacking specks might occur too.

**Figure 2:**
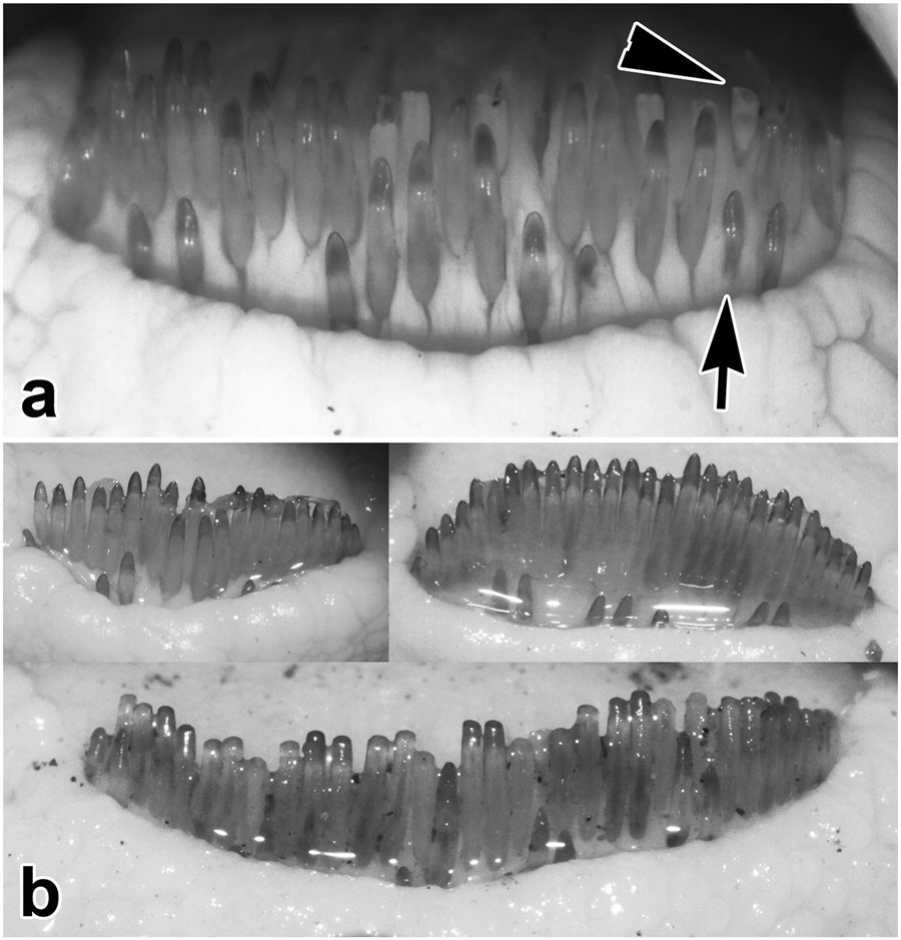
*Synodontis schall* mandibular dentition, Lake Turkana, Kenya. (**a)** High magnification image showing new teeth emerging at the outer margin of the tooth line (arrow, only one marked), main row of fully grown teeth with sharp tips (unmarked), and old teeth that already lost their sharp tips positioned at the inner margin of the tooth row (arrowhead, only one marked); (**b)** Composite image showing variability of teeth arrangement and number: 28 (upper left), 37 (upper right), 39 (bottom).

Standard length of examined *S*. *schall* specimens ranged from 142 to 300 mm (mean ± SD; 209.8 ± 33.2 mm). Weight ranged from 75 to 783 g (mean ± SD, 227.1 ± 118.9 g). *Synodontis schall* from the Nile and Turkana showed remarkable variation in body shape, proportions, as well as coloration and other qualitative diagnostic features (see Fig. 1 and Table 2 for details). We observed a phenotypic gradient from the typical *S*. *schall* morphotype (Fig. 1a,d,i,k), which dominated the sample, to the typical *S*. *frontosus* morphotype (Fig. 1c,h,j,m), which was less frequent. These observations, together with the common occurrence of intermediate forms hampered reliable morphology-based determination of numerous specimens. In the majority of Nile-Turkana specimens, there was an obvious, blunt, low, relatively wide, longitudinally striated keel on the ventral side of the humeral process fitting the *S*. *schall* type (Fig. 1d,e,h). Only in some specimens the keel was absent, resembling that of the *S*. *frontosus* type (Fig. 1f,g). The humeral process shape showed gradual variation from *S*. *schall*-like (margins straight and/or dorsally concave and/or ventrally convex) (Fig. 1d,e) to *S*. *frontosus*-like (straight both dorsally and ventrally) (Fig. 1h). Gradual variation was observed also in anterior pectoral spine serrations, from *S*. *schall*-like (fine, sharp, distinct) (Fig. 1i) to *S*. *frontosus*-like (barely discernible, sometimes due to pigmentation in dark-colored specimens) (Fig. 1j). Remarkable gradual variation was present also in maxillary barbels from *S*. *schall*-like (devoid of a basal membrane or with a hardly visible slat-like rudiment) (Fig. 1k,l) to *S*. *frontosus*-like (distinct, well developed dark basal membrane) (Fig. 1m). Mandibular teeth numbered from 26 to 46 in total; these teeth numbers were outside ranges of both *S*. *schall* (18–39) and *S*. *frontosus* (33–48) morphotypes (Table 2). When only a single (main) row of mandibular teeth was considered, the count was 21 to 40. Mandibular teeth appeared to have formed from the outer margin of tooth plate towards the mouth interior and the old ones lost their sharp tips and eventually disappear, while new ones start to grow from the outer side (Fig. 2). The number of new mandibular teeth emerging in front of the main teeth row varied from 1 to 12.

In three main Turkana sampling localities, the ratio of *S*. *schall*/*S*. *frontosus*/intermediate morphotypes was as follows: El-Molo Bay 35/16/7, Kalokol 19/11/0, Todonyang 32/31/14, implying somewhat higher relative incidence of *S*. *frontosus*-like morphotype in the freshwater part of Turkana (Chi-square test = 10.3; df = 4; P < 0.05) (Table S3; morphotype ratios for the Nile sample are not provided, because damage of fish from markets hampered assessment of all the focal features in some specimens).

In addition, several other features corresponding either to *S*. *schall* or *S*. *frontosus* have been observed, but not systematically recorded. For example, pointed snout combined with narrower interorbital region and eyes well within lateral margin of the head in dorsal view (as in *S*. *schall*) vs. snout more broadly rounded with wider interorbital region and orbits impinging on the lateral outline of the head in dorsal view (as in *S*. *frontosus*) (sensu Hopson^22^). The two states are well demonstrated by the two specimens showing *S*. *schall* and *S*. *frontosus* morphotype in Fig. 1a,c, respectively.

### Phylogenetic analyses

In total, 131 sequences of *coxI* (565 bp), and two smaller sets comprising 96 *cytb* (973 bp) and 19 *RAG2* (896 bp) sequences were generated. Data representing individual populations obtained from the Nile River and Lake Turkana were used for phylogenetic analyses together with *Synodontis* spp. sequences retrieved from GenBank. The newly generated sequence data were deposited in GenBank under accession numbers *coxI*: KY483652-KY483782, *cytb*: KY483783-KY483878, *RAG2*: MF136700-MF136718, MF150161- MF150164 (Table S1). As BI and ML phylogenetic analyses provided similar outcomes, only BI final trees are presented with posterior probabilities as branch supports, whereas ML trees are presented as Supplementary Data.

**Figure 3:**
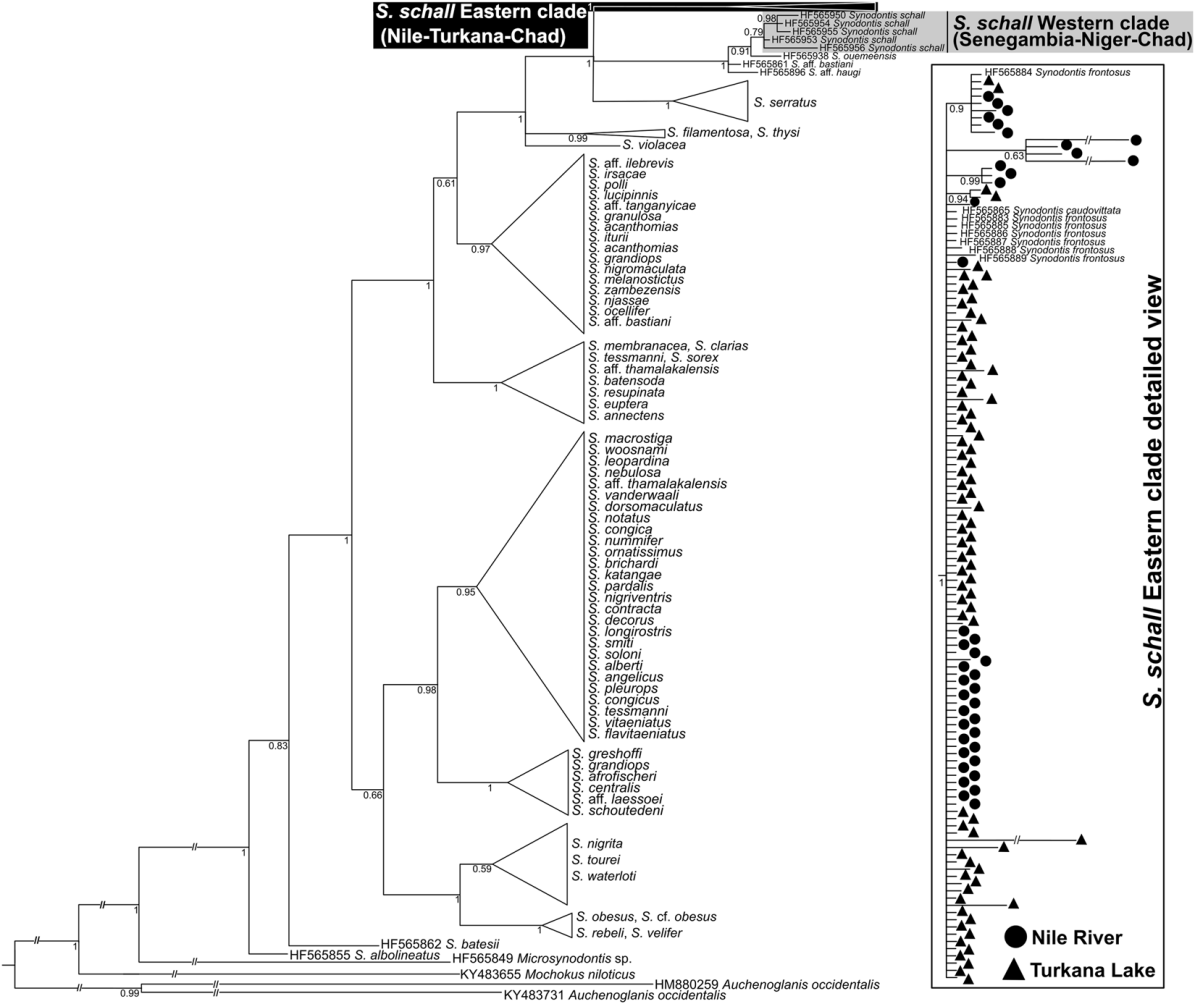
Bayesian phylogeny for *coxI*. Posterior probabilities are shown as branch supports. Tree is rooted with *Microsynodontis* sp. sequence. Samples of *S*. *schall* Eastern clade are collapsed; detailed view of Eastern clade structure is presented with sampling localities. Valid names of *Synodontis* spp. following^25^ are used for convenience. Original assignments retrieved from GenBank, together with sequence accession numbers are provided in Fig. S2 and Tables S1, S2.

In *coxI* analysis, sequences of 131 *Synodontis* spp. obtained for this study and 115 sequences from GenBank were used to construct the final tree (Fig. 3). As expected, our samples of *S*. *nigrita* and *S*. *serratus* from the Nile clustered with conspecific sequences from GenBank (see Fig. S1 for composition of the collapsed clades). All Nile-Turkana *S*. *schall* samples, i.e. *S*. *schall*-like, *S*. *frontosus*-like and intermediate morphotypes, clustered together and created a well-supported group. Samples from different Turkana or Nile localities did not show any geographical pattern or affiliation to their sampling localities. According to the predominant morphological patterns observed in the Nile-Turkana sample and location of the type locality of *S*. *schall* in the Nile, we named this clade “*S*. *schall* Eastern clade”. Importantly, all GenBank sequences labeled *S*. *frontosus* (HF565883–89) and one sequence of *S*. *caudovittatus* (HF565865), all from the Nile, also clustered within the Eastern clade in all analyses. In contrast, GenBank sequences labeled *S*. *schall* (HF565950, HF565953-56), *Synodontis ouemeensis* HF565953, all from western part of Nilo-Sudanian and Eburneo-Ghanian provinces, together with sequences labeled *Synodontis* aff. *bastiani* and *Synodontis* aff. *haugi* HF565950, HF565954-56 (pet trade), created a separate group with high nodal support. Due to the clear separation of this group from the “*S*. *schall* Eastern clade” and taxonomic assignment of most its sequences, we named the clade “*S*. *schall* Western clade”.

**Figure 4:**
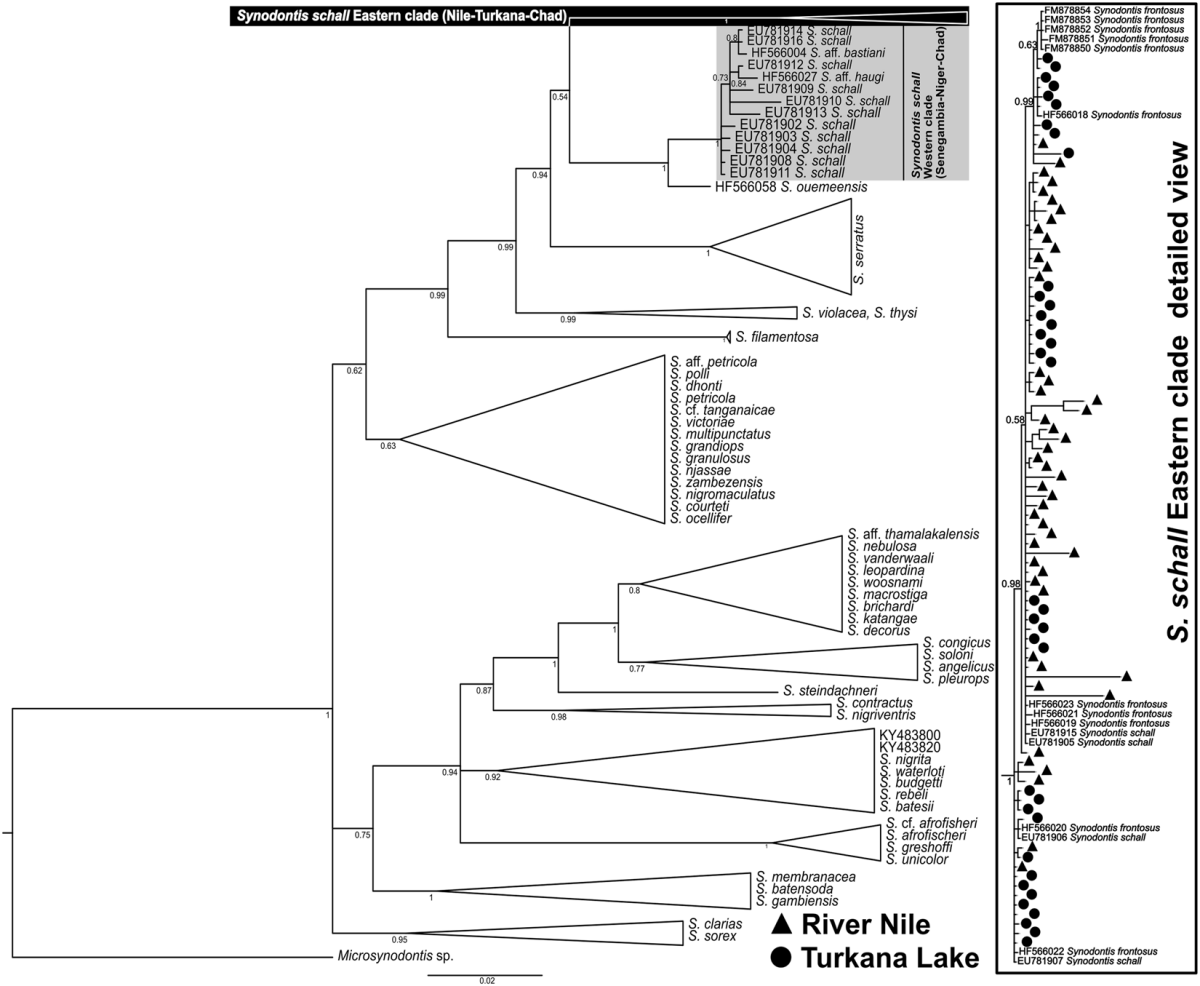
Bayesian phylogeny for *cytb*. Posterior probabilities are shown as branch supports Tree is rooted to *Microsynodontis* sp. sequence. Samples of *S*. *schall* Eastern clade are collapsed; detailed view of Eastern clade structure is presented with sampling localities. Valid names of *Synodontis* spp. following^25^ are used for convenience. Original assignments retrieved from GenBank, together with sequence accession numbers are provided in Fig. S2 and Tables S1, S2.

**Figure 5:**
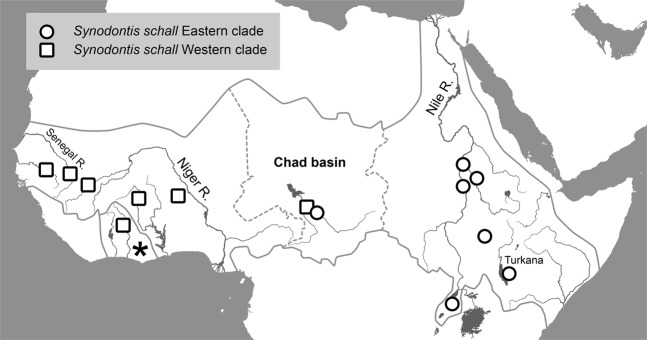
Map of the Nilo-Sudanian ichthyological province (solid grey line) showing geographical origin of *Synodontis schall* Eastern and Western clade samples as revealed by phylogenetic analyses. Dashed line indicates western and eastern limits of Chad basin; asterisk indicates Eburneo-Ghanian province; only major lakes are shown outside the area of interest.

The dataset for *cytb* included sequences representing 52 *Synodontis* spp. (Fig. 4). Our sequences clustered with all GenBank *S*. *frontosus* sequences (FM878850–54, HF566018–23), as well as *S*. *schall* sequences (EU781905–07, EU781915) and EU781915 from the Nile and/or Chad basin. This clade clearly corresponds with the *S*. *schall* Eastern clade, hence its name in the tree. The rest of *S*. *schall* sequences (EU781902–04, EU781908–14, EU781916) from Senegambia, Niger and Chad basin created a group corresponding to the *S*. *schall* Western clade, closely related to, but clearly distinct from the *S*. *schall* Eastern clade. Interestingly, *cytb* sequences of *S*. *ouemeensis* (HF566058), *S*. aff. *bastiani* (HF566004) and *S*. aff. *haugi* (HF566027) clustered with the *S*. *schall* Western clade data. This result is similar to *coxI* analysis, however; with a stronger branch support and placing sequences of *S*. aff. *bastiani and S*. aff. *haugi* within the Western clade. In conclusion, analyses of both mitochondrial markers revealed very similar patterns with strong biogeographic signals, indicating phylogenetic distinctiveness and geographic affinity of two clades, i.e. *S*. *schall* Eastern and *S*. *schall* Western clade (Figs 3–5).

Similarly as in the tree based solely on *cytb* sequences, the *S*. *schall* Eastern and *S*. *schall* Western clades were clearly separated from each other in the concatenated mtDNA dataset too (Fig. S3).

**Figure 6:**
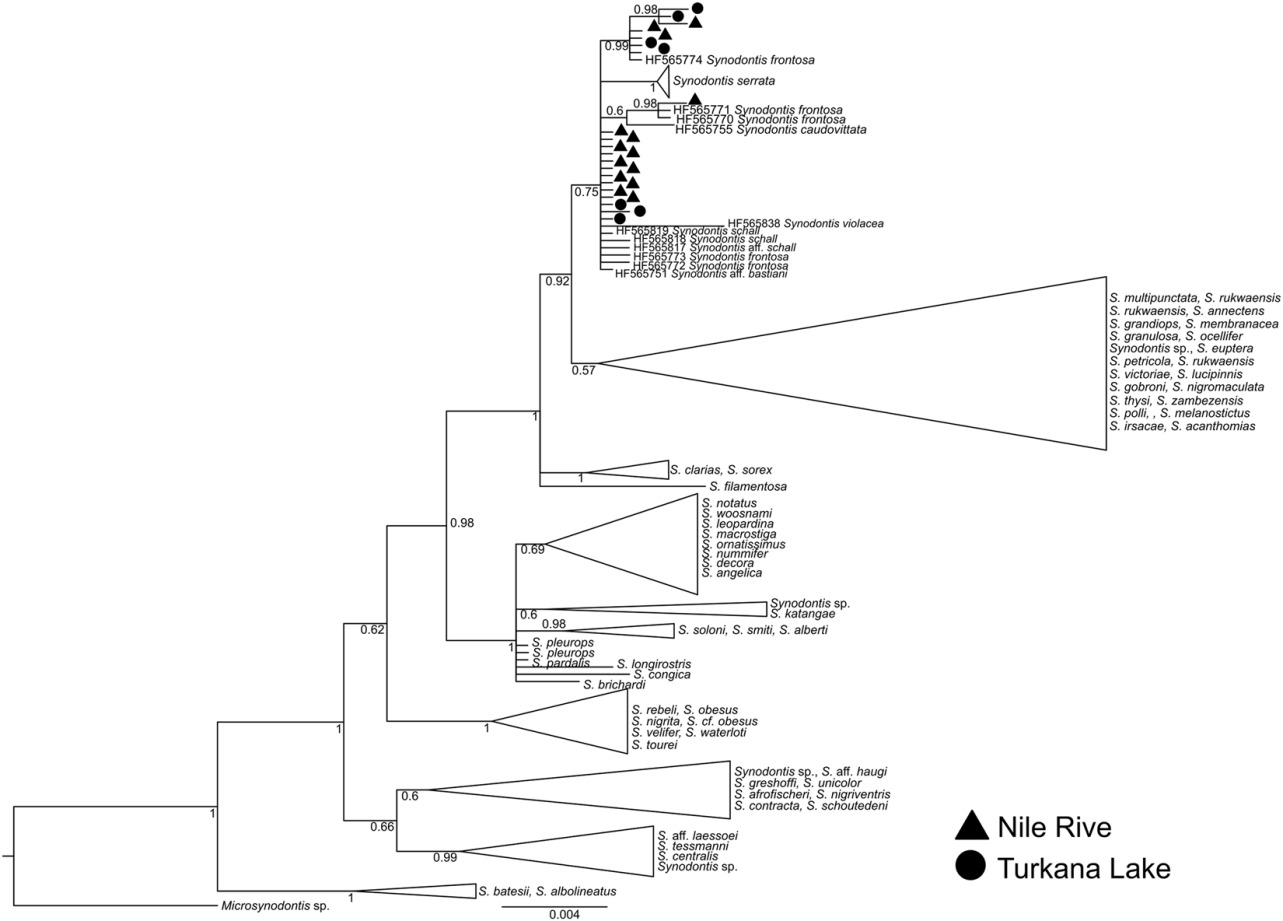
Bayesian phylogeny for *RAG2*. Posterior probabilities are shown as branch supports Tree is rooted with *Microsynodontis* sp. *Synodontis schall* Eastern clade is displayed in and branches for other *Synodontis* species are collapsed for better visualization. Valid names of *Synodontis* spp. following Froese & Pauly (2017) are used for convenience. Original assignments retrieved from GenBank, together with sequence accession numbers are provided in Fig. S3 and Tables S1, S2.

The phylogenetic analysis of the nuclear gene (*RAG2*) was less informative compared to the clear separation of Eastern and Western clades in all analyses performed for both mitochondrial genes. A total of 19 *RAG2* sequences of *Synodontis* spp. were analyzed (Fig. 6) together with 102 sequences from GenBank. Our *S*. *schall* Eastern clade samples from the Nile and Turkana comprised an internally unresolved cluster together with the samples of *S*. *frontosus* (HF565870-74), *S*. *caudovittatus* (HF565755), *S*. *violacea* (HF565838) and *S*. *aff*. *bastiani* (HF565751) from Eastern Nilo-Sudan province, as well as *S*. *schall* (HF565817-19) from Western Nilo-Sudan province. Although sequences of *S*. *serratus* from our sample and from GenBank clustered together with high support, the *S*. *serratus* branch was placed within the *S*. *schall* clade, probably as a result of low sequence variability in the whole dataset (100-94.5%).

### Genetic distance

Genetic distances between *S*. *schall* Eastern clade and Western clade samples range from 5.5% to 6.4% and from 5.7% to 8.3% for *coxI* and *cytb*, respectively (Table 3). In addition, the difference between the two *S*. *schall* clades is very similar to the distance between morphologically and molecularly well distinguished species *S*. *nigrita* and *S*. *serratus* (from 9.6% to 10.4%, see Table 3). The *coxI* data showed smaller range of variability, which might be due to the shorter sequence of the gene or simply, *coxI* is more conservative than *cytb*. Nevertheless, the distinction between *S*. *schall* clades is still more than 5% for both *coxI* and *cytb* mitochondrial genes.

**Table 3:** Genetic similarity between *coxI* and *cytb* sequences of *S*. *schall* Eastern clade, *S*. *schall* Western clade, *S*. *serratus* and *S*. *nigrita*.

coxI	S. schall Western clade	S. schall Eastern clade	S. nigrita	S. serratus
S. schall Western clade	x			
S. schall Eastern clade	94.5–93.6%	x		
S. nigrita	90.5–89.5%	90.4–89.1%	x	
S. serratus	94.4–92.5%	95.8–93.2%	90.4–89.6%	x
**cytb**				
	S. schall Western clade	S. schall Eastern clade	S. nigrita	S. serratus
S. schall Western clade	x			
S. schall Eastern clade	94.3–91.7%	x		
S. nigrita	91.2–90.4%	90.4–88.4%	x	
S. serratus	94.6–92.5%	93.5–92.3%	90.0–88.7%	x

### Haplotype networks

**Figure 7:**
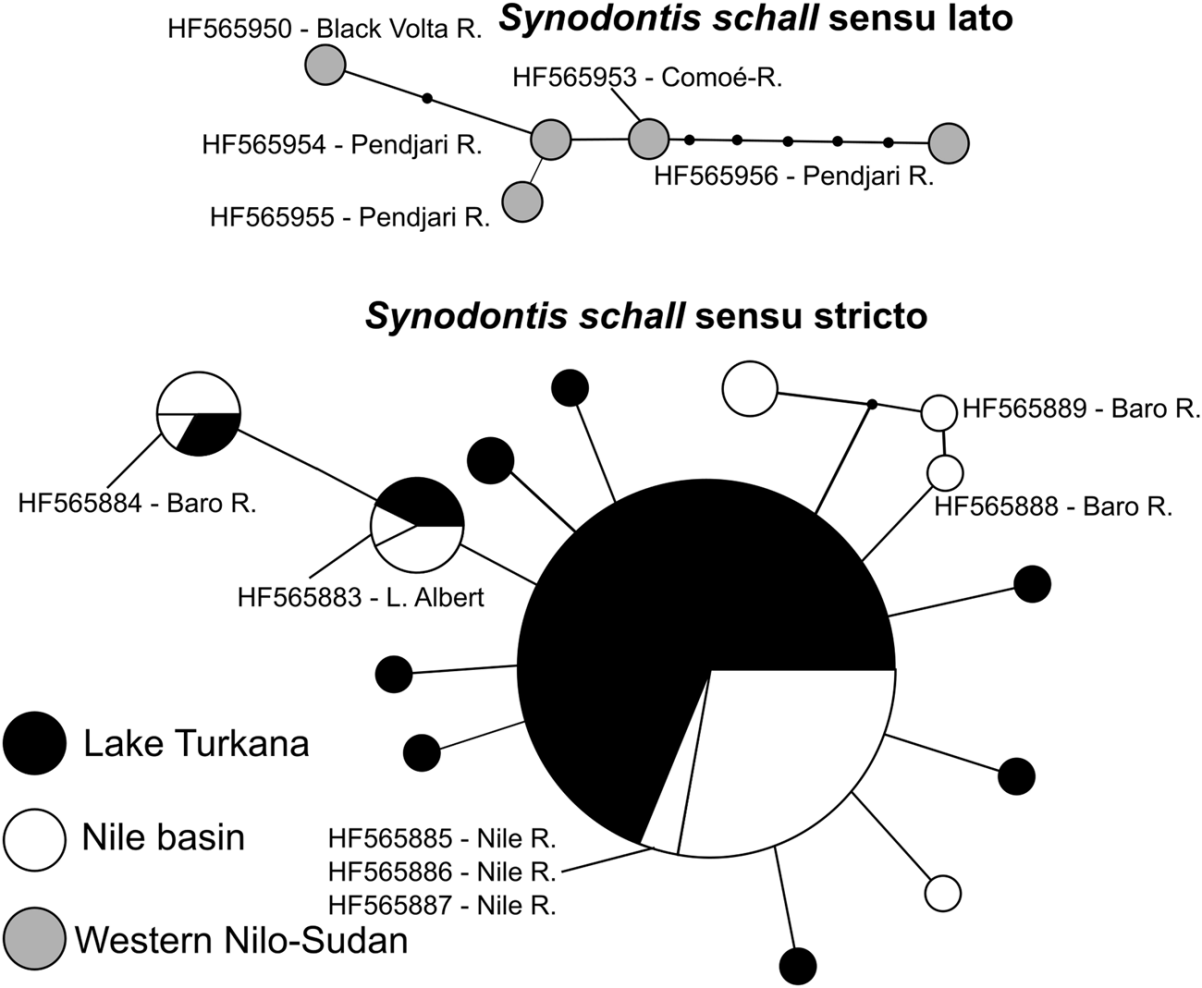
Haplotype networks for *coxI* constructed in software PopART v1.7. Networks were constructed only for samples clustering in *S*. *schall* Eastern and Western clades. Sizes of haplo-nodes are relative to the sample size. The two networks were separated by 28 steps. Accession numbers for GenBank sequences are listed in the picture along with sampling locations.

**Figure 8:**
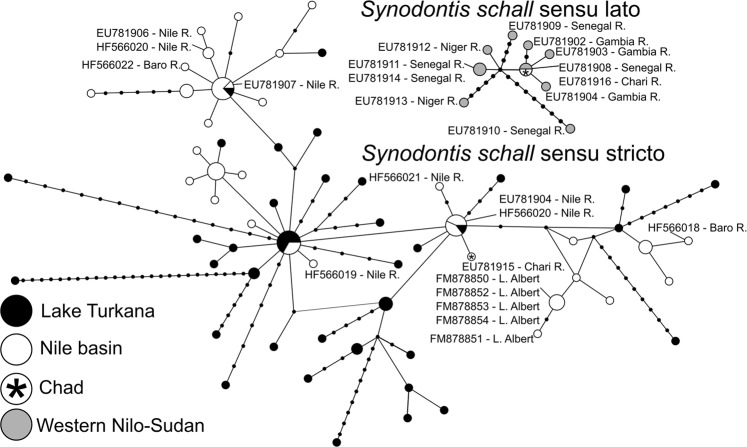
Haplotype networks for *cytb* constructed in software PopART v1.7. Networks were constructed only for samples clustering in *S*. *schall* Eastern and Western clades. Sizes of haplo-nodes are relative to the sample size. The two networks were separated by 51 steps. Accession numbers for GenBank sequences are listed in the picture along with sampling locations.

The *S*. *schall* Western clade produced a relatively compact network, exhibiting similar levels of diversity in both genes (Figs 7 and 8), with only a slight increase of distances between individual haplotypes in *cytb*. *CoxI* data for the Eastern clade also created a compact network, represented by one big central haplotype surrounded by derived haplotypes (Fig. 7), whereas *cytb* showed a more complicated network structure with some haplotypes divided by more than ten mutations (Fig. 8). Although the two Eastern clade networks might appear very different, similar phenomena can be seen in both datasets, most remarkably the sharing of haplotypes between the Nile and Turkana basins. The differences between structures of haplotype networks are probably due to a longer stretch of *cytb* sequence analyzed and a putatively higher mutation rate of *cytb* compared to *coxI* in vertebrates^50^.

### Population genetic statistics of the Eastern clade

Population genetic statistics and genetic distances performed for our data did not show profound differences between the sequences of *S*. *schall*-like and *S*. *frontosus-*like specimens belonging to the Eastern clade (see Table S4). Genetic distances between the individuals of the same respective morphotype were similar to the distances between individuals belonging to different morphotypes (approximately 0 to 1% and 0 to 2% for *coxI* and *cytb*, respectively, see Table S4). The overall F_ST_ values between the two morphotypes were 0.08 and 0.04 for *coxI* and *cytb*, respectively. The summed chi-square values, for the test of genetic differentiation, were moderately significant for *coxI* sequences (0.001 < *P* < 0.01) and non-significant for *cytb* (*P* > 0.05). Similarly, a difference was seen in haplotype diversities for *coxI*, but not in *cytb* (Table S4). Moreover, the statistics of genetic differentiation between localities within Turkana (*Snn test*, Table S4) revealed a significant value for *coxI* sequences, but not in *cytb*. Results of all these tests provide evidence of low level of the genetic differentiation corresponding to an intraspecific level.

## Discussion

### Identity of S. schall-like catfish

In the Nile and Turkana basins, typical morphotypes of both *S*. *schall* and *S*. *frontosus*, as well as a phenotypic gradient between the two, have been identified. Regardless of morphological patterns, phylogenetic and haplotype analyses suggest that all the Nile-Turkana samples are conspecific, including both *S*. *schall*-like and *S*. *frontosus*-like specimens. It is to be noted, that the taxonomical incongruence between taxonomic assignment of GenBank sequences as identified by other authors e.g. Day *et al*.^18^, Pinton *et al*.^19^, Day *et al*.^36^ and supported by our phylogenetic analyses of *coxI* and *cytb* data clearly indicated both *S*. *schall* and *S*. *frontosus* morpho-species among samples revealed herein as conspecific (see original sequence assignments retained in Fig. 4 and Table S2). This result casts doubt on features presumably differentiating *S*. *schall* and *S*. *frontosus*. Especially, the relatively frequent presence of distinct dark membranes on maxillary barbels because its “absence, or a presence of only hardly visible rudiment” (sensu Paugy & Roberts^21^ and Musschoot & Lalèyè^17^) was considered a basic feature distinguishing *S*. *schall* from *S*. *frontosus*. The genetic uniformity of the Nile-Turkana sample on one hand, and its remarkable morphological variability lead us to assume that diagnostic features of *S*. *frontosus* merely describe part of the intraspecific variability of *S*. *schall*. In other words, explicit evidence of remarkable intraspecific variability provided herein renders the two nominal species from East Africa, *S*. *schall* and *S*. *frontosus*, most probably synonymous, hence requiring taxonomical reassessment. Additionally, from a strictly phylogenetic point of view, the clustering of *Synodontis* sequences assigned to several species (*S*. *ouemeensis*, *S*. aff. *bastiani*, *S*. aff. *haugi* and *S*. *schall*) implies that these data might represent a single species *S*. *schall* Western clade, especially considering uncertain status of many *Synodontis* nominal species.

Based on the following criteria, we assign the Nile-Turkana population, i.e. *S*. *schall* Eastern clade, as represented in our sample to *S*. *schall* sensu stricto: (i) the most prevalent morphotype matching *S*. *schall*, (ii) phylogenetic affinity (see below), (iii) geographic origin matching with the type locality in the Nile and (iv) with respect to probable synonymy, the specific name *S*. *schall* has priority over *S*. *frontosus*. The interpretation is further indirectly supported by the supposed absence of *S*. *frontosus* in Lake Turkana itself, being presumably limited to Omo River (as highlighted by Hopson 1982 and other authors). Although in our Turkana sample *S*. *frontosus* morphotype was not restricted to the freshwater Omo delta, it was most prevalent here, whereas the *S*. *schall* morphotype was the most prevalent in the Turkana saline and medium salinity parts. Differences in the prevalence of morphotypes between the localities may also coincide with the moderate (intraspecific) level of genetic differentiation found in the population genetic analysis of *coxI* (*Snn* test, Table S4). The *coxI* pattern is probably caused by an ongoing process of lineage sorting. No evidence for morphotype or genetic differentiation was found in the genetically more diverse *cytb*.

Mitochondrial data revealed no significant differentiation between specimens representing *S*. *schall* and *S*. *frontosus* morphotypes, indicating their possible conspecificity. However, it should be noted that an alternative evolutionary scenario could generate such a genetic pattern - a complete fixation (or replacement) of mitochondrial DNA introgressed from one species to another. Unfortunately, we were not able to corroborate the mitochondrial results by an independent marker. The phylogeny based on the nuclear *RAG2* gene was not informative, due to the lack of resolution in the respective part of the tree. Lack of representative data from the Western Nilo-Sudanian province, together with the generally low variability of the gene did not allow clear distinction of mitochondrial lineages in the nuclear dataset. Although generally rare, complete fixation of introgressed mitochondrial DNA was found for example in African cichlids^51–53^ and coral reef fish^54^. Analyses of more variable nuclear loci (e.g. introns or whole genome analyses) would be required to completely exclude such a possibility in *S*. *schall*-like catfishes. However, considering the weak differentiation of morphological traits defining *S*. *schall* and *S*. *frontosus*, as opposed to the high level of *cytb* diversity required to be introgressed between the two species across all sampled localities, we consider the “introgression and fixation” alternative to be much less probable than conspecificity.

Concurrent morphological and molecular sampling throughout the Nile basin, including terra typica of both *S*. *schall* and *S*. *frontosus*, i.e. Nile River in Assouan and in Khartoum, respectively, is necessary for sound taxonomical measures (we presume synonymy of *S*. *frontosus* and *S*. *schall*). Unfortunately, studies of other authors do not aid further resolution, because even recent taxonomic studies^17,20,37,38^ on Nilo-Sudanian *Synodontis* spp. lack molecular phylogenetic analyses, and vice versa, all available phylogenetic studies lack clues for the determination of sampled specimens, and/or the exact origin of the samples^17,28^.

### Hidden diversity of S. schall-like catfish of the Nilo-Sudanian ichtyological province

The combined Nile-Turkana and GenBank dataset comprises all major limnic systems of the Nilo-Sudanian province, i.e. rivers Gambia, Senegal, Volta, Niger, Nile (including Lake Albert), endorheic basins of Chad and Turkana, as well as Eburneo-Ghanian (sub) province. As such, the presented dataset allows us to draw conclusions regarding diversity of *S*. *schall*-like catfish throughout the Nilo-Sudanian province. The results of the phylogenetic reconstruction, haplotype analyses and genetic distance recognized different *S*. *schall* clades. Phylogenetic analyses of both mitochondrial markers unequivocally revealed separate *S*. *schall* Eastern and Western clades, each comprising a mixture of samples assigned by different authors to *S*. *schall*, *S*. *frontosus* or other taxa. In addition to that, the genetic similarities between *S*. *schall* Eastern and *S*. *schall* Western clades sequences showed more than 5% divergence for both mitochondrial genes, a phenomenon generally associated with taxa recognized as distinct species^55^. Regardless of GenBank taxonomic sequence assignment, the two well supported clades, *S*. *schall* Eastern and Western clade, exclusively comprise either samples from eastern part of the Nilo-Sudanian ichthyological province (Nile, Turkana, Chad), or those from its western part (e.g. Senegambia, Niger, Chad), respectively. The taxonomically incongruent, but geographically non-random phylogenetic pattern reveals a strong biogeographical signal among the supposedly homogenous, conspecific and widely distributed *S*. *schall*-like catfish.

*Synodontis schall* was long considered morphologically uniform and widely distributed throughout the Nilo-Sudanian ichtyological province from the Atlantic to Indian Ocean^17,21^. In contrast, our results imply the presence of two relatively distant phylogenetic lineages hidden under this name. *Synodontis schall* sensu stricto, as defined by location of type locality in the Nile, corresponds to the Eastern clade in our analyses, and is restricted to the province’s eastern part, i.e. Nile, Turkana and Chad basins, and possibly elsewhere in north-east Africa (Fig. 6). On the other hand, *S*. *schall*-like catfish from the western part of the Nilo-Sudanian province (*S*. *schall* Western clade in the present study) probably represent a cryptic species more closely related to congeners from the same region than to their eastern siblings. Interestingly, there seems to be an overlap in distribution ranges of the “Eastern clade” and the “Western clade” in the Chad basin, probably as a result of independent and temporally distinct colonization events.

The phenomenon of cryptic, geographically determined diversity in African fish described herein is not a new discovery. A similar pattern was described for African tigerfish genus *Hydrocynus*^56^. The cryptic diversity, i.e. presence of three lineages among *Hydrocynus vittatus* populations from three separate drainages (Zambezi, Congo and Okavango), was revealed based on a combination of morphological and molecular (*cytb*) data. Similarly, as in *S*. *schall*-like catfishes in this study, the diversity of *H*. *vittatus* has long been overlooked due to the absence of definitive morphological features and molecular evidence. Another possible example of overlooked diversity among African fish, but revealed morphologically in this case, might be two cyprinid species, *Labeo niloticus* and *L*. *senegalensis*. The two *Labeo* spp. show a distribution pattern almost identical to that of *S*. *schall*-like catfishes are also distinguished by subtle, sometimes overlapping morphological features. Morphological studies including samples from western (*L*. *senegalensis*) and eastern (*L*. *niloticus*) parts of Nilo-Sudan ichthyological province revealed apparent hidden diversity correlated with geographical origin of specimens^19,57^. However, molecular data are not yet available for *L*. *senegalensis* and *L*. *niloticus* and their species status remains to be confirmed. The multiple examples of overlooked diversity in *H*. *vittatus* and *Labeo* spp., as well as *S*. *schall*-like catfish herein, suggest that the phenomenon of hidden diversity among long known and geographically widely distributed African fishes might be more common than previously thought.

## Conclusions

*Synodontis schall* (Bloch et Schneider, 1801), as it has been defined and recognized for more than two centuries, apparently includes two distinct phylogenetic units (Eastern and Western clades), i.e. two distinct species are hidden within this name. The Eastern clade represents *S*. *schall* sensu stricto sensu stricto as defined by the type locality in the Nile, while the Western clade from the western part of Nilo-Sudanian province probably represents a cryptic species, which remained unrecognized due to a lack of differentiating morphological traits. A candidate name *Synodontis gambiensis* Günther, 1864 (Terra typica: Gambia), currently considered a junior synonym of *S*. *schall*, is available for eventual taxonomical amendment of the Western clade. In contrast, another long-recognized nominal species, *S*. *frontosus* Vaillant, 1895, might be merely a junior synonym of *S*. *schall* sensu stricto. An interesting, rather unusual taxonomical case has emerged from this study, since concurrent taxonomical deflation (nominal species synonymisation) and taxonomical split (cryptic species formal recognition) might be inevitable in the future for the two long recognized congeners.

## Supplementary information

Supplementary info

Supplementary Tables S1, S2, S3, S4
