# Author Correction: From taxonomic deflation to newly detected cryptic species: Hidden diversity in a widespread African squeaker catfish

**DOI:** 10.1038/s41598-019-56348-4

**Published:** 2019-12-20

**Authors:** Dagmar Jirsová, Jan Štefka, Radim Blažek, John O. Malala, David E. Lotuliakou, Zuheir N. Mahmoud, Miloslav Jirků

**Affiliations:** 1 Faculty of Science, University of South Bohemia, Branišovská 1760, 370 05 České, Budějovice Czech Republic; 2 Institute of Parasitology, Biology Centre, Czech Academy of Sciences, Branišovská 31, 370 05 České, Budějovice Czech Republic; 3 Institute of Vertebrate Biology, Czech Academy of Sciences, Květná 8, 603 65 Brno, Czech Republic; 4 Department of Botany and Zoology, Faculty of Science, Masaryk University, Kotlářská 2, 611 37 Brno, Czech Republic; 5 Kenya Marine and Fisheries Research Institute, Lake Turkana Station, P.O. Box 205, 30500 Lodwar, Kenya; 6 Department of Zoology, Faculty of Science, University of Khartoum, 111 15 Khartoum, Sudan

Correction to: *Scientific Reports* 10.1038/s41598-019-52306-2, published online 31 October 2019

This Article contains errors in Figures 7 and 8 where the labels *Synodontis schall* sensu lato and *Synodontis schall* sensu stricto are incorrect. The correct Figures 7 and 8 are displayed below as Figures 1 and 2.

**Figure 1:**
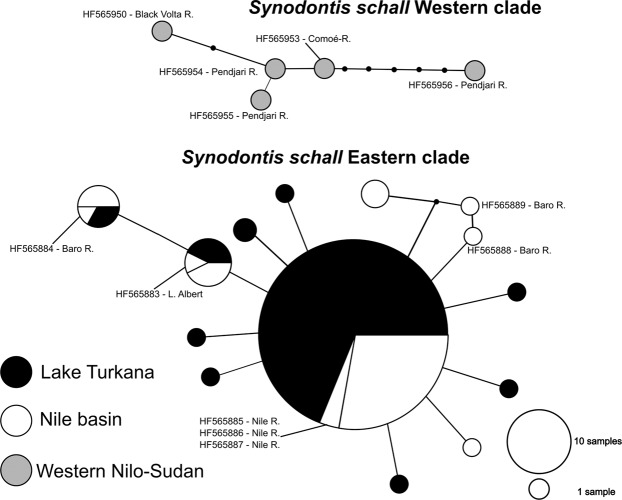
.

**Figure 2:**
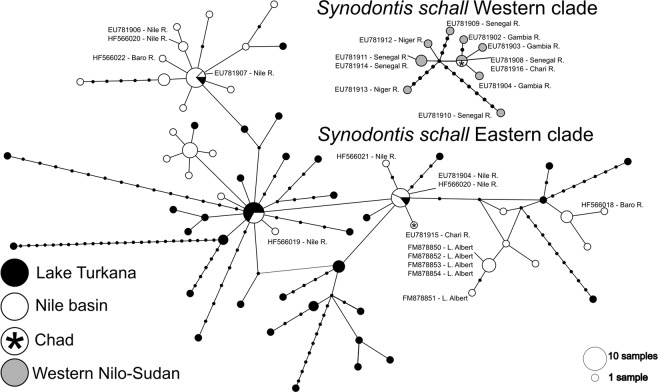
.

