# Supplementary material for: From taxonomic deflation to newly detected cryptic species: Hidden diversity in a widespread African squeaker catfish
Source: Sci Rep. 2019 Oct 31;9:15748. doi: 10.1038/s41598-019-52306-2 (PMC6823466; doi:10.1038/s41598-019-52306-2)
Supplement: Supplementary file 1 — Supplementary info [file 41598_2019_52306_MOESM1_ESM.pdf]

From taxonomic deflation to newly detected cryptic species: Hidden diversity in a widespread African squeaker catfish

Dagmar Jirsová[1,2]*, Jan Štefka[1,2], Radim Blažek[3,4], John O. Malala[5], David E. Lotuliakou[5], Zuheir N. Mahmoud[6], Miloslav Jirků[2]

Supplementary data

Table S1. Sequences of *coxI*, *cytb* and *RAG2* for *Synodontis* spp. from the Nile and Turkana deposited in GenBank, including detailed information on localities. Data provided in separate file.

Table S2. List of GenBank *coxI*, *cytb* and *RAG2 Synodontis* spp. sequences used in phylogenetic and haplotype analyses Data provided in separate file. The data obtained from the same sample are highlighted by bold.

Table S3. Results of chi-square test for spatial distribution of *S. schall*, *S. frontosus* and intermediate morphotypes.

obs.		El Molo	Kalokol	Todonyang	totals(marginals)
	1	35	19	32	86
	2	16	11	31	58
	3	7	0	14	21
		58	30	77	165

obs. frekv.		El Molo	Kalokol	Todonyang	null (expected)
	1	0,603	0,63	0,415	0,52
	2	0,276	0,36	0,402	0,35
	3	0,121	0	0,181	0,13

expected counts		El Molo	Kalokol	Todonyang	
	1	30,23	15,63	40,13	
	2	20,38	10,54	27,06	
	3	7,38	3,81	9,8	

		El Molo	Kalokol	Todonyang	
χ2		0,753	0,723	1,648	
		0,944	0,019	0,571	
		0,019	3,818	1,8	
		1,716	4,561	4,019	**10,297**
**P(=0,05; df=4)**		**9,488**			

Table S4. Results of population genetics analyses between *S. schall*-like and *S. frontosus*-like morphotype from Eastern Africa (the Nile and Turkana) obtained for this study. The genetic differentiation between the sequences from three Turkana localities was tested by the *Snn* test and showed that the saline locality differs from the freshwater and medium part.

	**Haplotype diversity**			
	coxI	*cytb*		
S. schall-like	0.36	0.97		
S. frontosus-like	0.65	0.97		
	**Fst**			
	coxI	*cytb*		
S. schall-like x *S. frontosus*-like	0.08	0.04		
	**Genetic differentiation**			
	coxI		*cytb*	
	Chi-square	*P*	*Chi-square*	*P*
S. schall-like x *S. frontosus*-like	34.487	0.0029	51.934	0.1922
	**Genetic distance**			
	coxI	*cytb*		
S. schall-like	0 - 0.88 %	0 - 1.75%		
S. frontosus-like	0 - 1.06%	0 - 1.95%		
S. schall-like x *S. frontosus*-like	0 - 1.06%	0 - 2.06%		

**Turkana localities**							
	**Haplotype diversity**						
	coxI	*cytb*					
Medium salinity	0.38	1.00					
Freshwater	0.40	0.98					
Saline water	0.67	0.93					
	**Fst**						
	coxI				*cytb*		
	Medium salinity	Freshwater	Saline water		Medium salinity	Freshwater	Saline water
Medium salinity	x			Medium salinity	x		
Freshwater	0.00585	x		Freshwater	0.063	x	
Saline water	0.18779	0.17877	x	Saline water	0.047	0.010	x
	**Snn test**						
	coxI	*cytb*					
Snn	0.68	0.543					
P-value	0.018	0.094*					
* not significant P-value							

a

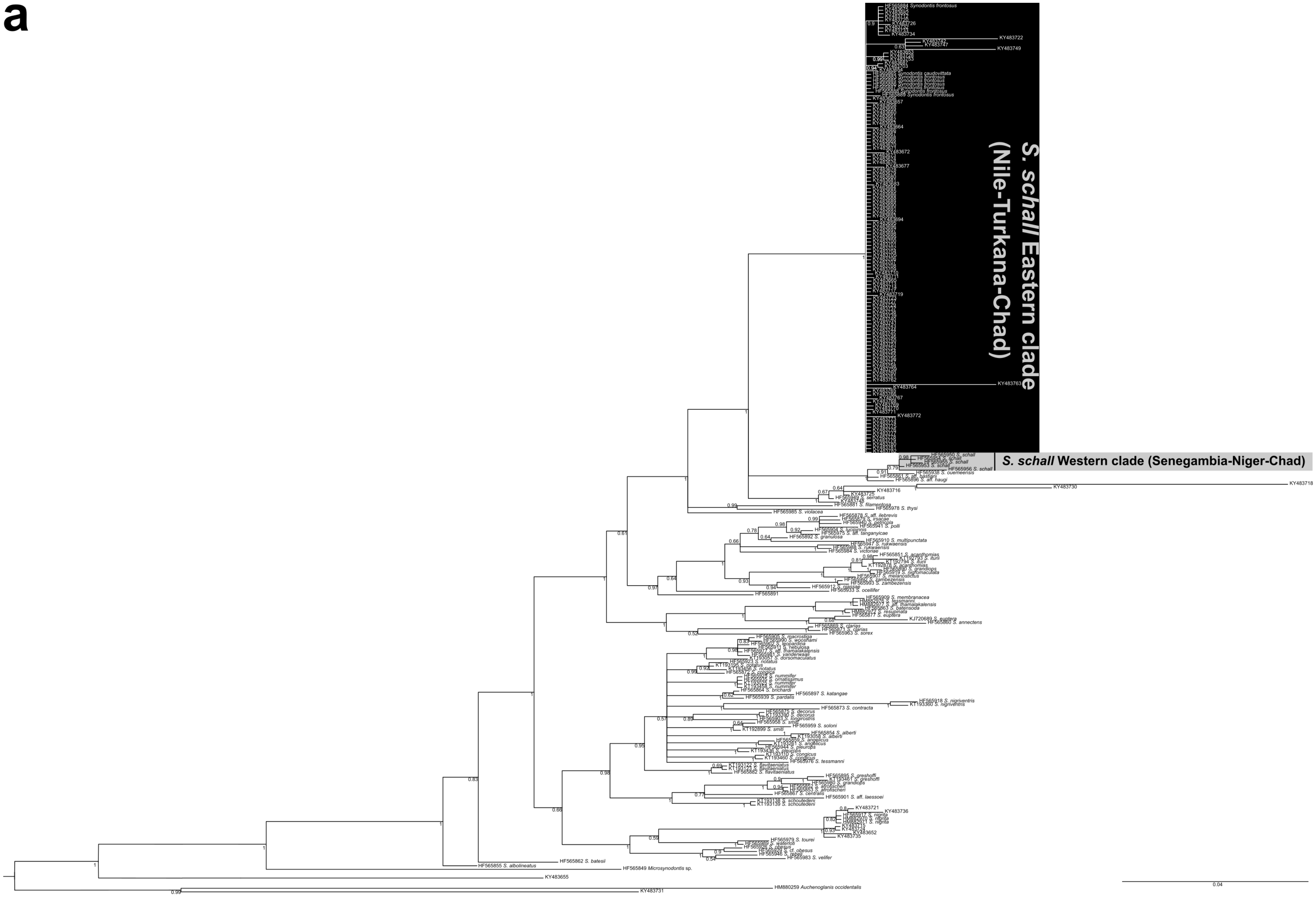

S. schall Eastern clade
(Nile-Turkana-Chad)
S. schall Western clade (Senegambia-Niger-Chad)
0.04

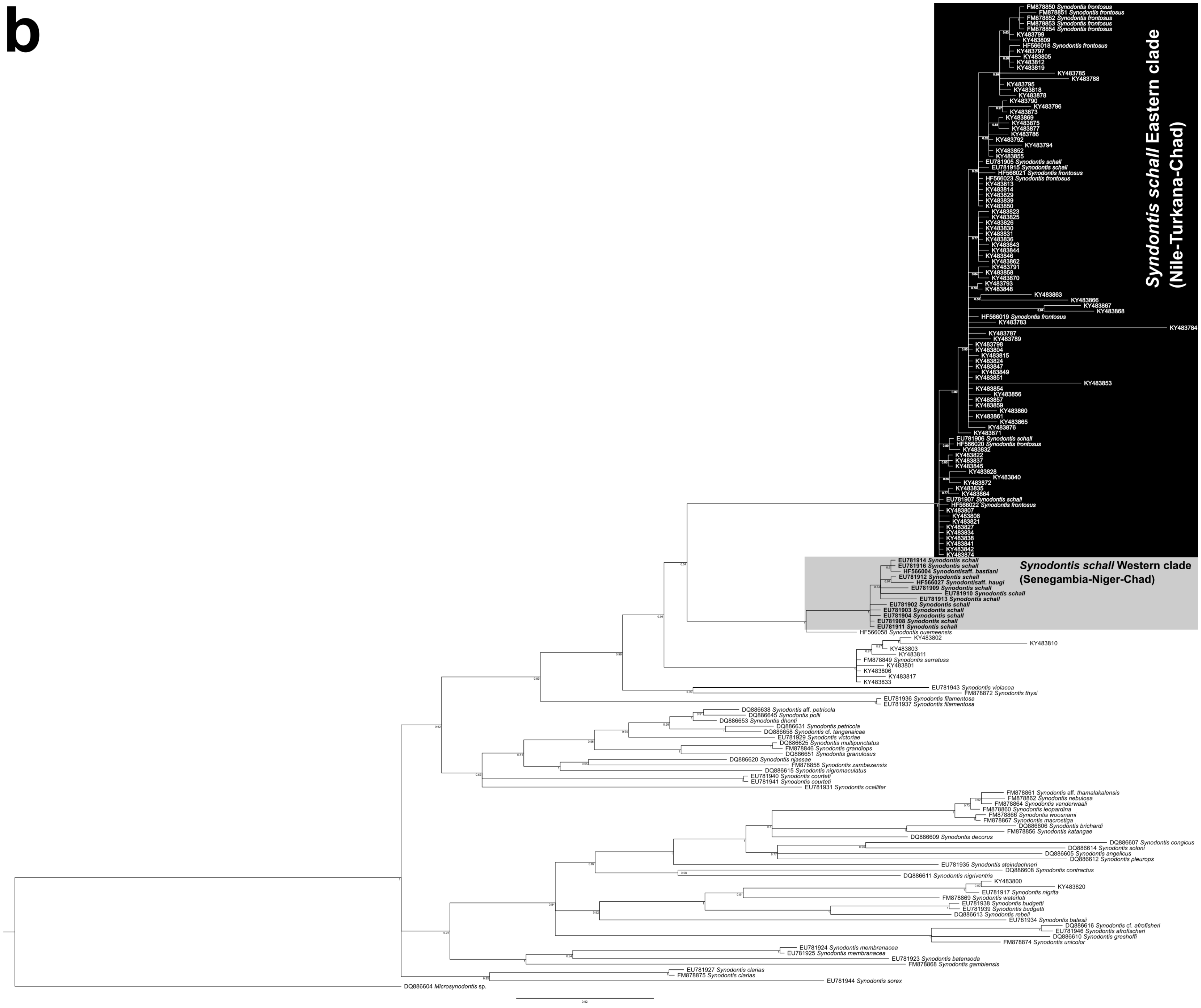
b
Syndontis schall Eastern clade
(Nile-Turkana-Chad)
Synodontis schall Western clade
(Senegambia-Niger-Chad)

c

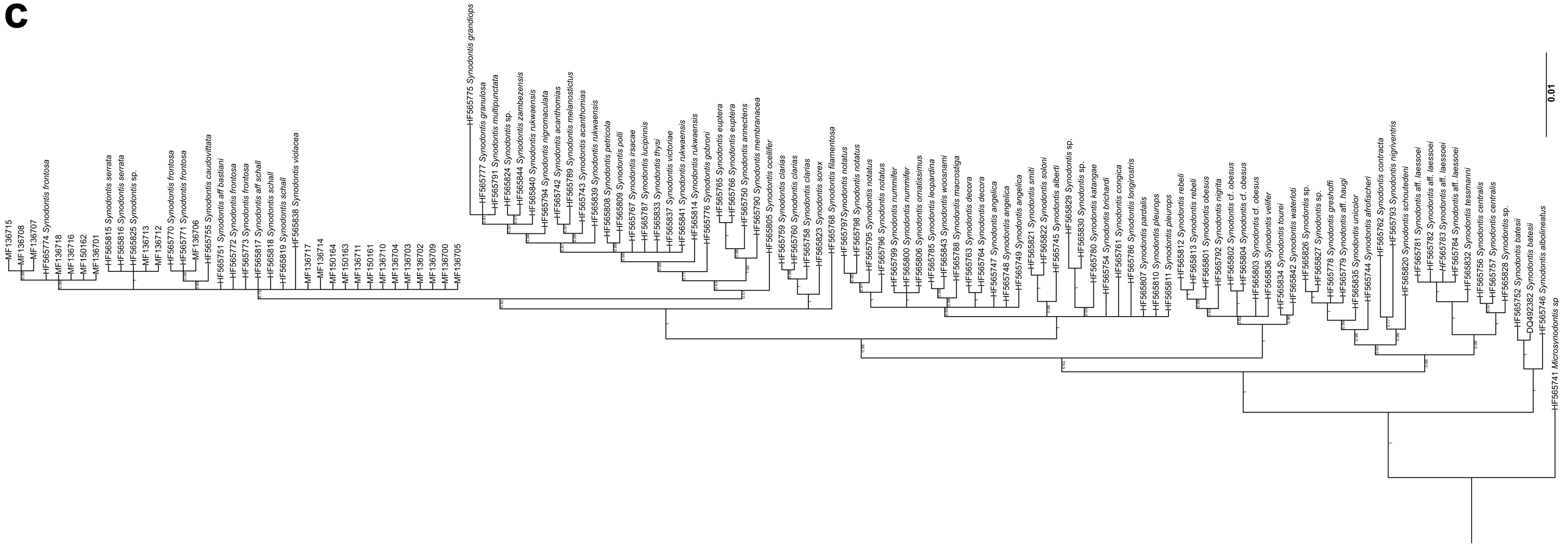

Figure S1. Phylogenetic trees for *coxI* a), *cytb* b) and *RAG2* c) as revealed by BI analyses, showing detailed branching with posterior probabilities as branch supports. Final trees were created by software MrBayes. Sequence data of *S. schall* Eastern clade obtained within the scope of this study are presented with GenBank numbers. Data provided in separate file (see Tab. S2).

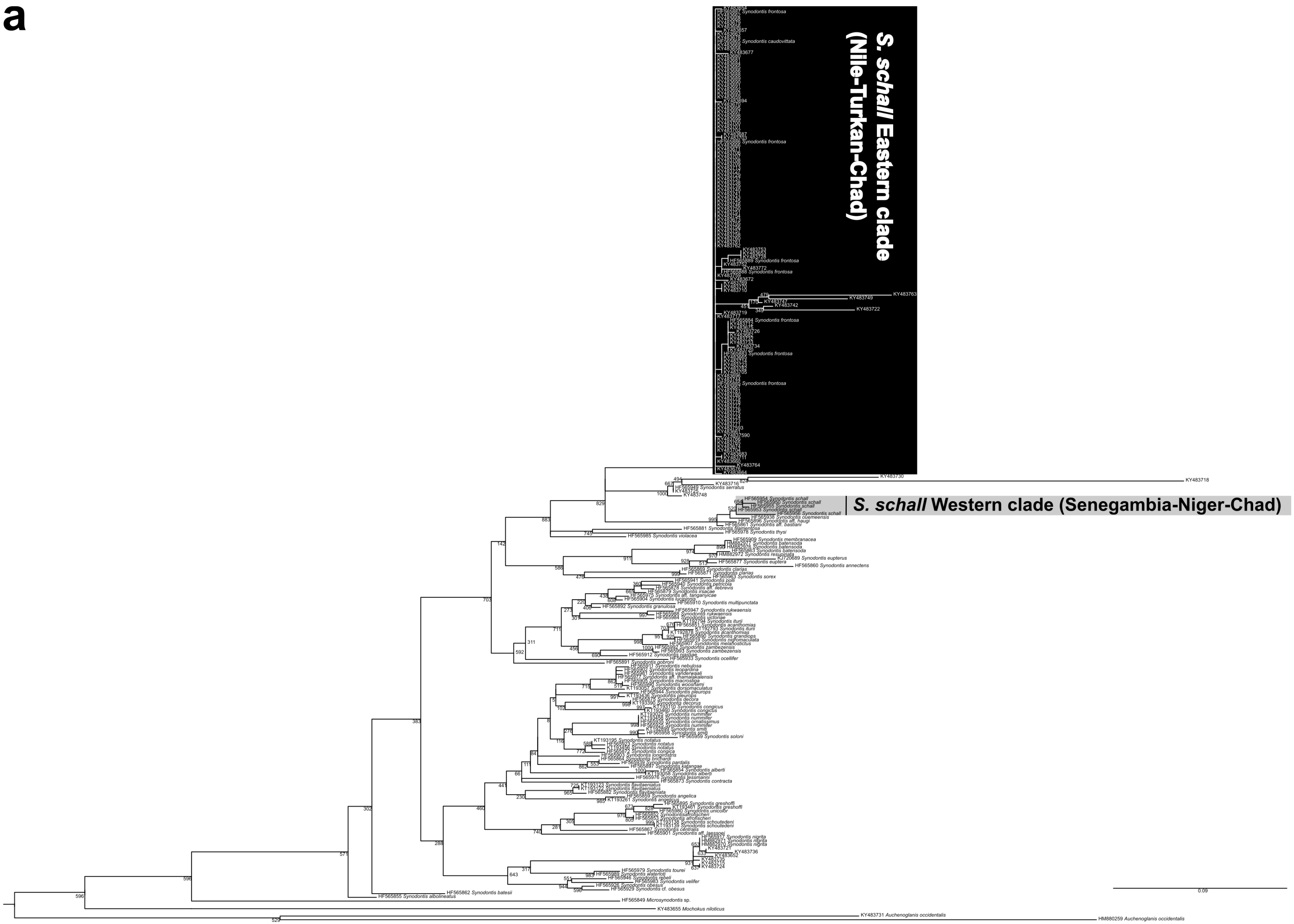
a
S. schall Eastern clade (Nile-Turkan-Chad)
S. schall Western clade (Senegambia-Niger-Chad)
0.09

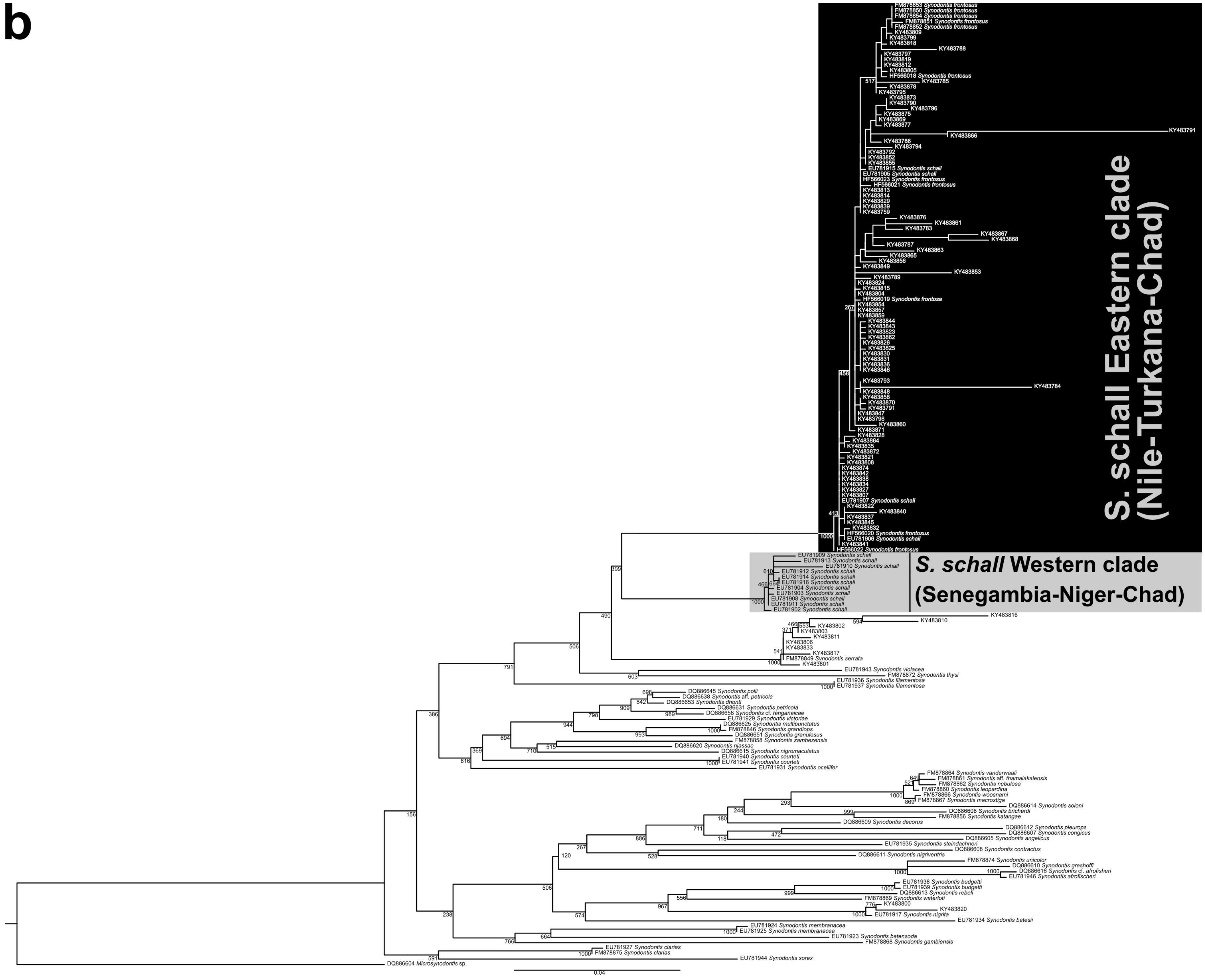
b
S. schall Eastern clade
(Nile-Turkana-Chad)
S. schall Western clade
(Senegambia-Niger-Chad)

c

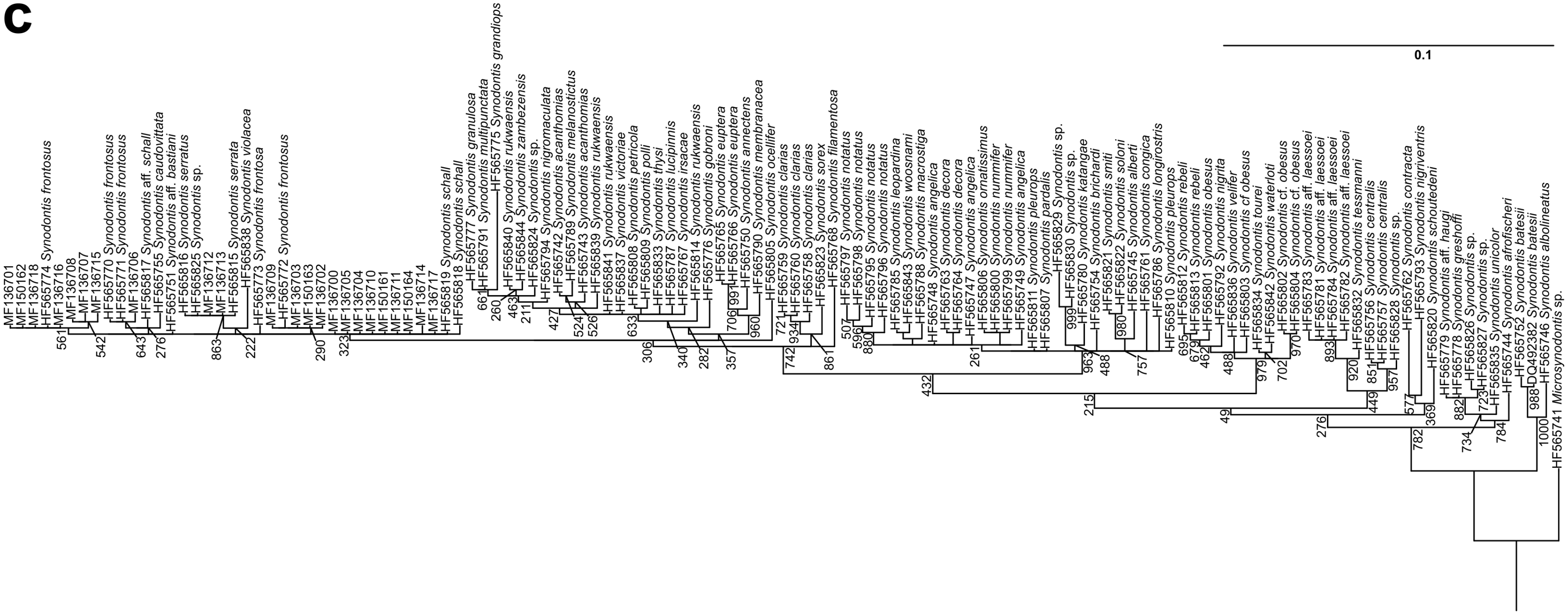

Figure S2. Phylogenetic trees for *coxI* a), *cytb* b) and *RAG2* c) as revealed by ML analyses, showing detailed branching with bootstraps as branch supports. Sequence data of *S. schall* Eastern clade obtained within the scope of this study are presented with GenBank numbers (see Tab. S2).

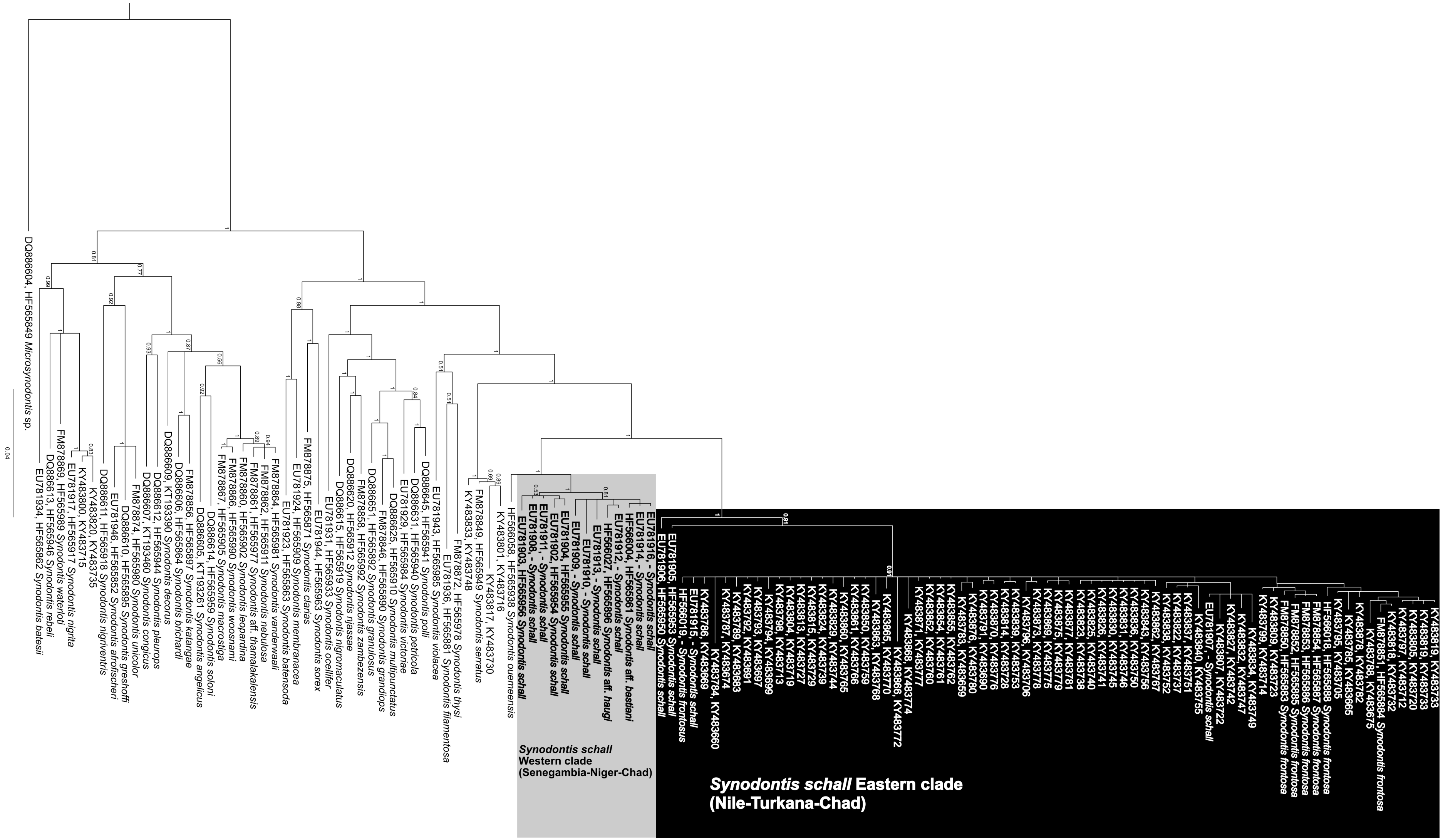

Figure S3. Phylogenetic analysis of concatenated dataset for mt genes (*coxI* and *cytb*) as revealed by BI analyses, showing detailed branching with posterior probabilities as branch supports. Final trees were created by software MrBayes. Sequence data of *S. schall* Eastern clade obtained within the scope of this study are presented with GenBank numbers. Data provided in separate file (see Tab. S1 and S2).

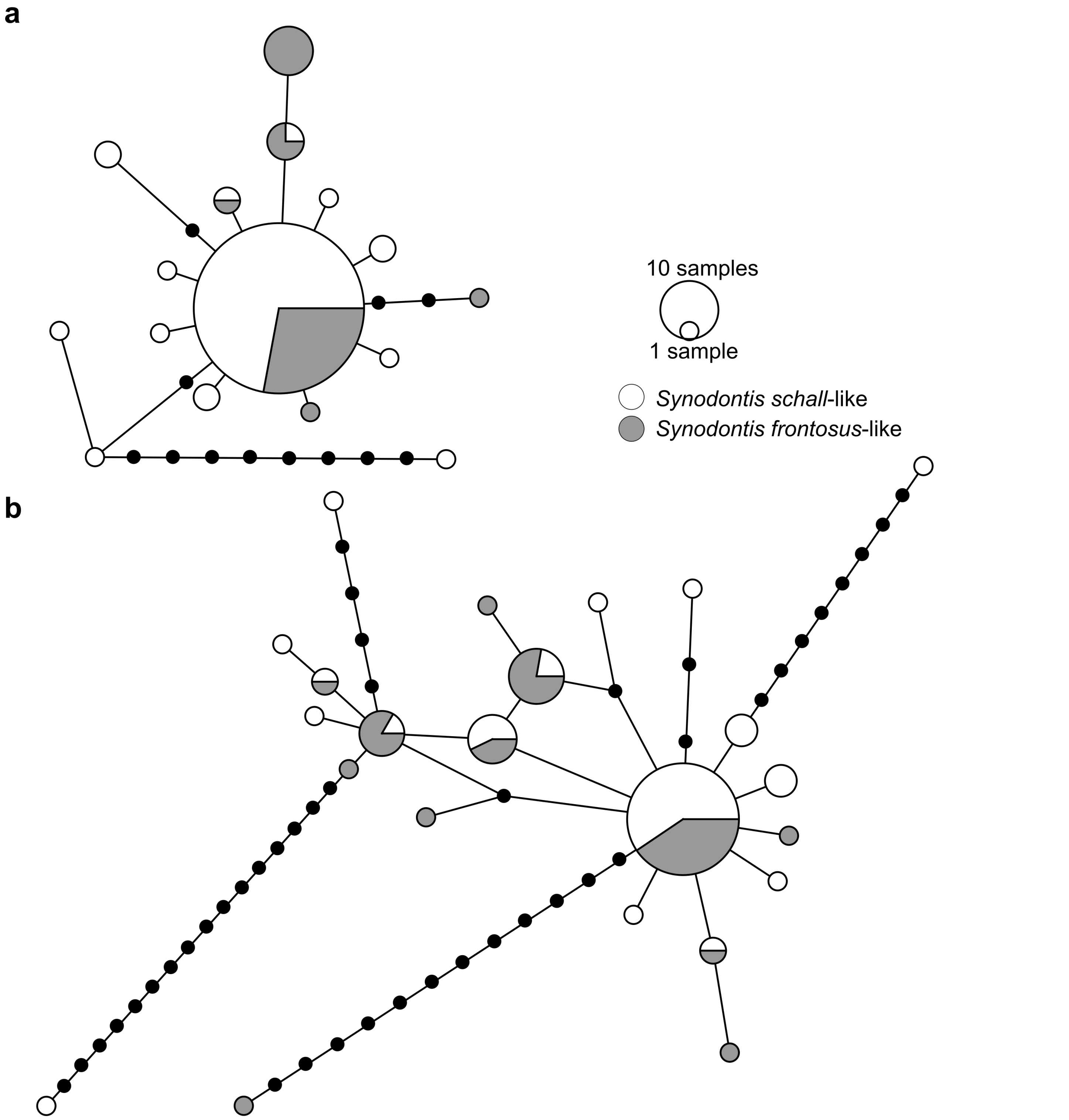

Figure S4. Haplotype networks for *coxI* (a) and *cytb* (b) constructed in software PopART v1.7. Networks were constructed only for samples obtained for this study. Sizes of haplo-nodes are relative to the sample size.
